# Two-dimensional mass defect matrix plots for mapping genealogical links in mixtures of lignin depolymerisation products

**DOI:** 10.1007/s00216-016-9598-5

**Published:** 2016-05-14

**Authors:** Yulin Qi, Rolf Hempelmann, Dietrich A. Volmer

**Affiliations:** Institute of Bioanalytical Chemistry, Saarland University, Campus B2.2, 66123 Saarbrücken, Germany; Institute of Physical Chemistry, Saarland University, Campus B 22, 66123 Saarbrücken, Germany

**Keywords:** Lignin, High resolution mass spectrometry, Kendrick mass, Mass defect, 2D matrix plot, FTICR-MS

## Abstract

Lignin is the second most abundant natural biopolymer, and lignin wastes are therefore potentially significant sources for renewable chemicals such as fuel compounds, as alternatives to fossil fuels. Waste valorisation of lignin is currently limited to a few applications such as in the pulp industry, however, because of the lack of effective extraction and characterisation methods for the chemically highly complex mixtures after decomposition. Here, we have implemented high resolution mass spectrometry and developed two-dimensional mass defect matrix plots as a data visualisation tool, similar to the Kendrick mass defect plots implemented in fields such as petroleomics. These 2D matrix plots greatly simplified the highly convoluted lignin mass spectral data acquired from Fourier transform ion cyclotron resonance (FTICR)–mass spectrometry, and the derived metrics provided confident peak assignments and strongly improved structural mapping of lignin decomposition product series from the various linkages within the lignin polymer after electrochemical decomposition.

**Electronic supplementary material:**

The online version of this article (doi:10.1007/s00216-016-9598-5) contains supplementary material, which is available to authorized users.

**Graphical Abstract:**
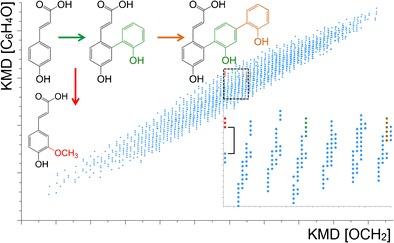
2D mass defect matrix plot for a lignin sample after decomposition

## Introduction

The massive worldwide consumption of petroleum and the limited future availability of fossil resources have prompted the development of alternative biofuels. Storable liquid biofuels greatly reduce greenhouse gas emissions in comparison to conventional fossil fuels [1, 2]. While traditional biofuels are mostly produced from vital food supplies such as sugar cane and corn starch, alternative sources—in particular agricultural and municipal wastes—are now increasingly important to recover valuable chemicals contained in the chemically complex wastes. Importantly, these chemicals have significant value beyond simple energy generation.

Lignin is a major component of the cell wall of woody plants and the second most abundant natural biopolymer after cellulose [3]. Studies have shown that the chemical content of lignin is similar to that of fossil fuels [4]. Unfortunately, commercial applications of lignin have remained limited and focus mainly on low value products (e.g. raw materials for chemicals; dispersants for cement dyes; or dust suppression agents for roads) [2, 5], leaving lignin highly underutilised as alternative to conventional biofuels and source of high value chemicals [6].

**Fig. 1:**
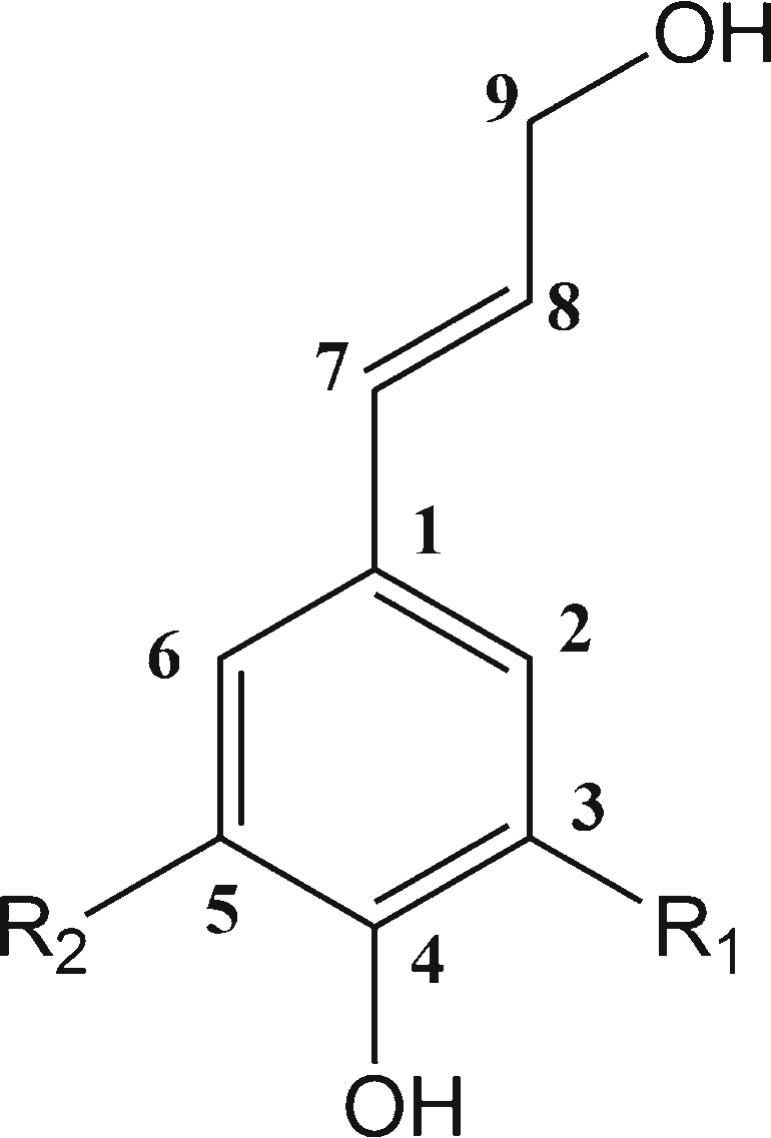
Structures of the three main lignin monolignols with numbering scheme for the carbon positions: coumaryl alcohol (R_1_ = R_2_ = H), coniferyl alcohol (R_1_ = OCH_3_, R_2_ = H), and sinapyl alcohol (R_1_ = R_2_ = OCH_3_)

Structurally, lignins are cross-linked macromolecules with molecular weights of up to 10,000 Da [7], which are mostly formed via free radical coupling of three basic hydroxyphenylpropanoid monolignols: coumaryl alcohol, coniferyl alcohol and sinapyl alcohol (Fig. 1). Catalysed by oxidative enzymes, electrons are delocalised in these monolignols, providing unpaired electron density at positions 1, 4, 5 and 8. The 8-position is the most favoured among the various linkages (Fig. S1, Electronic Supplementary Material, ESM) and the dominant bond in the lignin polymer is therefore the 8-O-4 linkage, comprising approximately 50 % of the linkages in the woody plants [8].

Because lignin polymers are complex heterogeneous materials, the full characterisation of the individual components at the molecular level is required to assess and salvage the desired high value chemicals from these polymers. Various degradation, extraction and catalysis methods have been developed to explore the lignin content [9, 10]. Unfortunately, the different methodologies often result in different estimates of lignin products, even from the same sample [11], as a result of lignin’s inherent complex structural motifs and variable relative abundances within the material. Fortunately, modern instrumental analytical techniques have improved the structural identification process. For example, nuclear magnetic resonance (NMR) was utilised to analyse lignin from plant cell wall [12, 13], which was helpful to describe the different building blocks and bond types of lignin compounds. NMR requires relatively large amounts of analyte material from purified or concentrated samples, however. As a result, NMR experiments of lignin only revealed average structural features of the bulk mixtures, while information on low abundant, uncharacterised building blocks could not be determined [14, 15].

Mass spectrometry (MS) provides important complementary information to NMR, which has been readily demonstrated for samples such as crude oil. Full mass scan spectral analysis of crude oil samples using ultra high resolution Fourier transform ion cyclotron resonance - mass spectrometry (FTICR–MS) generated thousands of *m*/*z* features per sample, with relative abundances stretching over several orders of magnitude [16, 17]. From these mass spectra, the individual compounds’ accurate masses and elemental formulae were readily determined [18–20]. But even though each peak represented a chemically distinct compound, their structures mostly remained unknown. Lignin exhibits a similarly complex set of components, which has been coined the “lignome” [21]. The lignome is defined as the complement of all phenolics, which are regulated as part of the lignin biosynthesis [22]. Consequently, lignomics analysis of products formed from lignin decomposition and structural interpretation of lignin oligomers are similarly complicated as the petroleomics analysis of crude oils, requiring significant efforts for liquid chromatography (LC) separation, tandem mass spectrometry (MS/MS) and high resolution mass spectrometry (HRMS) analyses [22–27]. A very promising approach was presented by Morreel et al., who successfully elucidated the gas-phase fragmentation behaviour of lignome-associated molecules using comparative MS^*n*^ analyses [22]. The authors were able to assign characteristic fragmentation reactions for the essential lignin-bonding types and obtained diagnostic ions for the characteristic aromatic units, providing a foundation for detailed characterisation of the lignome and for MS-based sequencing of lignin oligomers. Owen et al. developed an LC method for lignin degradation products, which was able to analyse lignin-related model compounds in negative ion electrospray ionisation (ESI) mode, giving elemental compositions from accurate mass measurements by FTICR, followed by detailed MS^3^ experiments for structural information. The authors demonstrated the utility of their method using a mixture of lignin degradation products [27].

Unfortunately, these and other HRMS full-scan and MS/MS structural protocols are not well suited for obtaining detailed identification of thousands of individual components in heterogeneous mixtures from lignin decomposition. Therefore, data simplification and classification tools are required to reduce the complexity and to visualise the hidden information in the complex mass spectra, to better characterise and classify the samples.

For the study of natural organic matter (NOM), various graphical and statistical methods have been developed to simplify high resolution mass spectra (HRMS). For example, Kendrick mass defect (KMD) analysis has allowed the alignment of thousands of peaks across the *m*/*z* range according to their homologous structural units [28]. The van Krevelen diagram helps in sorting the same classes of compounds in specific locations within the diagram and makes it possible to investigate possible links between molecules [29, 30]. Double bond equivalence (DBE) and carbon number distribution plots have been utilised to examine the structural features of natural organic matter in samples from different origins [31].

In this work, lignin was depolymerised by electrochemical decomposition [32] and the products measured by FTICR–MS to obtain detailed insight into the composition of the chemically complex degradation mixtures. In recent work, we demonstrated the proof-of-concept of using HRMS for these mixtures and investigated appropriate ionisation techniques for analysis [33]. We also applied KMD and van Krevelen plots to interrogate the full-scan mass spectra, and observed a first glance of the enormous complexity of the mass spectral data sets. Here, we are significantly expanding the investigation by implementing much more advanced concepts for data mining of the lignin decomposition products. From the mass spectral raw data, more than 5000 elemental compositions were assigned in a single full-scan mass spectrum. As a result of isobaric inferences, however, most of the low abundant species were impossible to isolate for detailed MS/MS structural analyses, even though they were clearly visible in the high resolution mass spectra. In order to process the bulk of the features contained in the data sets, the concept of two-dimensional (2D) fractional mass filtering, derived from KMD plots, was utilised as a visualisation tool to allow meaningful interpretation of the observed signals and compounds. The 2D matrix plots provided systematic line-ups of the different lignin linkages using structure-specific metrics. The procedure greatly simplified data interpretation, because candidate *m*/*z* values and chemical structures were readily deduced from the genealogical links between products and their formation mechanisms, rather than unsystematically assigned chemical formulae in conventional elemental composition analysis. Starting from the low *m*/*z* region—consisting mainly of monomers—the core structures of lignin were readily identified via MS/MS; the higher oligomer structures originated from the same linkages and were therefore quickly visualised according to the mass defect base applied in the matrix plots. Structures of higher molecular weights—but lower abundances in the samples—could then be predicted on the basis of this information, and the full-scan data sets specifically mined for them. For validation, the proposed structures were confirmed by detailed collision-induced dissociation (CID) experiments.

## Experimental

### Reagents and chemicals

Methanol, ammonium hydroxide and the alkali lignin standards were purchased from Sigma-Aldrich (Steinheim, Germany). The electrochemical degradation process was performed as described previously [32, 33], yielding 20 % solid phase (w/w, based on mass of lignin precursor). The powder was dissolved in water/methanol/ammonium hydroxide (50:50:1 v/v/v) prior to MS analysis. Organic-free water was generated by a Millipore (Bedford, MA, USA) Direct-Q8 purification system.

### High resolution mass spectrometry

Samples were ionised using electrospray ionisation (ESI) in negative ion mode. Mass spectra were recorded using a 7-Tesla FTICR–MS instrument (Bruker, Bremen, Germany) equipped with an Infinity cell [34]. For each spectrum, 40 individual transients were collected and co-added to the enhance signal-to-noise ratio (S/N) [35]. In MS/MS mode, precursor ions were isolated first in the quadrupole and externally accumulated for 0.1–1 s. For CID, 5–30 V collision voltage was applied.

### Data processing

The acquired mass spectra were loaded into Bruker DataAnalysis 4.2 software for data interpretation. The full-scan mass spectra were internally calibrated using a series of homologous compounds throughout the *m*/*z* range from 121 to 731. Elemental formulae were assigned to the peaks inside the calibrated *m*/*z* range, with the following tolerances to filter the lignin compounds: composition was restricted to C, H and O; DBE ranged from 4 to 25; H/C ratios from 0.6 to 2, and O/C ratios from 0.2 to 0.7 were considered; the acceptable mass error was ±1.5 ppm for singly charged ions. The assigned mass values were imported into an Excel spreadsheet to rescale the *m*/*z* regions of interest.

## Results and discussion

Electrochemical decomposition of alkali lignin was performed as described previously [32]. In the current study, negative ion electrospray ionisation (ESI) was implemented for MS analysis for two reasons: (1) the lignin decomposition compounds contain hydroxyl (–OH) groups, which were readily amenable to deprotonation to give [M−H]^−^ analyte ions; (2) ESI minimised sample consumption, which was particularly important for the detailed MS/MS acquisition routines performed here. Even though atmospheric pressure photoionisation (APPI) has been shown to provide slightly higher compound coverage in our preliminary experiments [33], the amount of sample required for APPI (or atmospheric pressure chemical ionization, APCI) was significantly higher, which was deemed unpractical in this study.

### General appearance of mass spectra and peak assignments

**Fig. 2:**
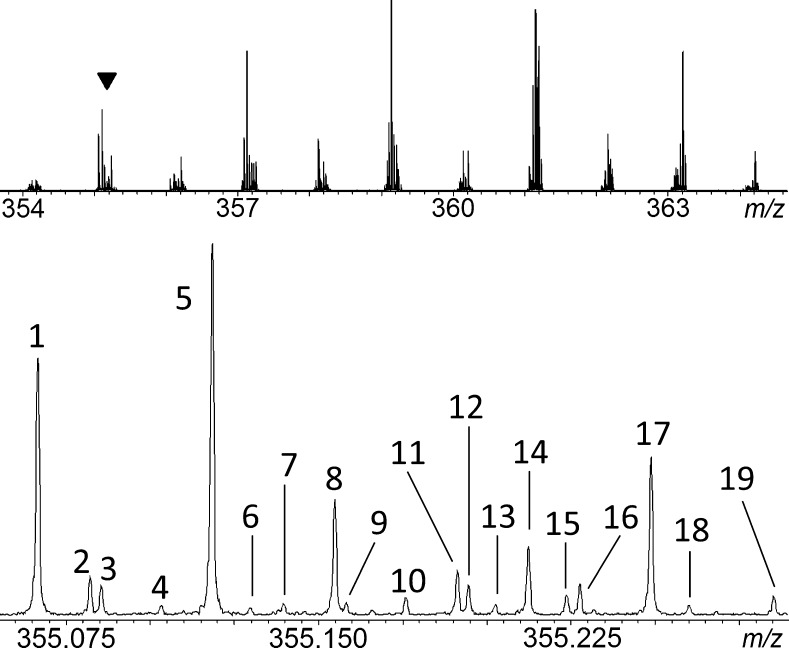
FTICR–MS full-scan analysis of lignin sample after electrochemical decomposition: (*top*) detailed view of mass segment *m*/*z* 354–364; (*bottom*) further mass scale-expansion of the region *m*/*z* 355.0–355.3 (marked with *inverted triangle* symbol in *top trace*; chemical formulae of these signals can be found in Table S1, ESM)

The electrochemically degraded lignin sample was initially measured in the full-scan mode of the FTICR instrument. The negative ion ESI–FTICR measurements revealed thousands of peaks in the mass spectrum with calculated resolutions (*m*/△*m*_50%_, FWHM) of greater than 400,000 at *m*/*z* approx. 300 (Fig. 2). Comparable complexities and peak densities have been observed in environmental samples such as crude oil, asphaltenes and humic acids [36–39]. To demonstrate the density of signals in the full-scan spectra, a region of 1 *m*/*z* unit was further expanded in Fig. 2, exhibiting a total of 19 assigned features in this small segment.

Throughout the spectrum, odd nominal *m*/*z* values dominated over even mass numbers. The limited nitrogen content in the lignin samples caused this bias, as zero N atoms in the elemental composition automatically gives an odd *m*/*z* ion according to the nitrogen rule. Moreover, many of the peaks at even *m*/*z* ratios were ^13^C [M+1] isotope species originating from odd *m*/*z* ions; they were readily confirmed by adding the mass of a neutron to the odd *m*/*z* species. A set of assignment criteria (viz. compositions restricted to C, H and O; DBE between 4 to 25; H/C ratios from 0.6 to 2; O/C ratios from 0.2 to 0.7; mass error max. ±1.5 ppm for singly charged ions) were then applied as a filter to exclude non-lignin signals, yielding approx. 2300 lignin-related compounds, which were further analysed. Even though high resolution FTICR–MS greatly helped in the unambiguous assignment of elemental compositions for these compounds, it was not possible to fully characterise their individual chemical structures, as detailed MS/MS analysis of each of these 2300 signals was not feasible. For this reason, post data processing methods were developed to reduce the complexity of the full-scan data, to enable rapid identification of important candidate components within the mixture for further analysis. In addition, linked *m*/*z* series were identified using this approach, from similarities and genealogical connectivities between species. These similarities and connections can illustrate different degradation pathways such as oxidation, demethylation, dehydration, dehydrogenation and aromatic ring cleavages.

### Two-dimensional matrix network

The Kendrick mass defect (KMD) [40] plot is useful approach to identify compound classes in crude oil samples [28]. In the original application of the KMD, the *m*/*z* scale was converted from 14.01565 u to 14 u using the repeating CH_2_ unit:

1 $$ \mathrm{Kendrick}\;m/z=\mathrm{IUPAC}\;m/z \times \left(14/14.01565\right) $$

It follows that compounds with the same number of heteroatoms and DBE, but different number of CH_2_ units, will have identical Kendrick mass defects:

2 $$ \mathrm{Kendrick}\kern0.28em \mathrm{mass}\kern0.28em \mathrm{defect}=\mathrm{nominal}\kern0.28em \mathrm{Kendrick}\kern0.28em m/z-\mathrm{exact}\kern0.28em \mathrm{Kendrick}\kern0.28em m/z $$

This rescaling process makes it possible to quickly recognise compounds of the same chemical class and type—but different extent of alkylation (CH_2_)—from the complex data sets. Importantly, KMD is not limited to CH_2_ groups; any functional group of interest can be used as the base for KMD:

3 $$ \mathrm{Modified}\;\mathrm{Kendrick}\ m/z=m/z\times \left(\mathrm{nominal}\ m/zof\;\mathrm{base}\ \mathrm{unit}/\mathrm{exact}m/z\kern0.2em \mathrm{of}\;\mathrm{base}\;\mathrm{unit}\right) $$

That is, KMD is applicable to any base unit or even higher-order mass defect analysis [33, 41, 42].

**Fig. 3:**
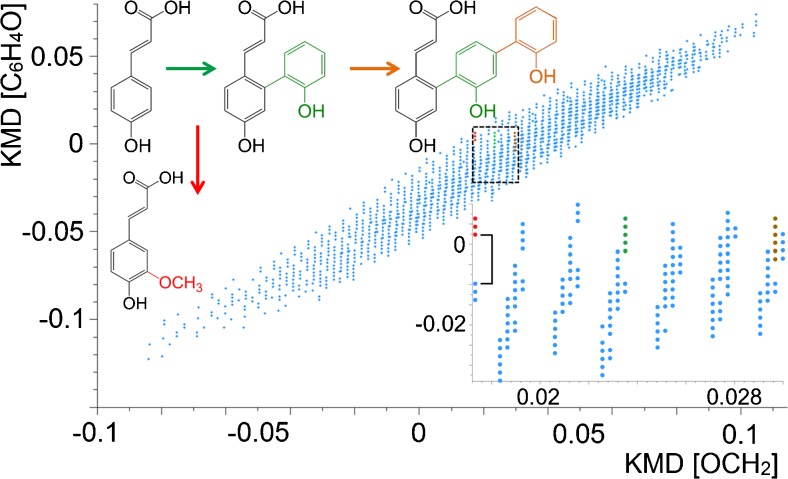
2D mass defect matrix plot for a lignin sample after decomposition. *Blue data points* represent features in the KMD plot and correspond to degradation products from the sample. The *squared area* is enlarged (*inset*) and proposed core structures of the three compound species (*red*, *green*, *brown*) are highlighted in the expanded plot

The first KMD model used here for lignin decomposition products was based on the simple coumaryl alcohol, with complex oligomers formed via methoxylation [OCH_3_] reactions, and various phenyl [C_6_H_4_O] linkages [32]. Therefore, these two structural units were chosen as KMD bases, to rescale the *m*/*z* axis of the mass spectra. The first modified KMD plot shown here used [OCH_2_] as horizontally rescaled *x*-axis (corresponding to methoxylation), while the phenol core [C_6_H_4_O] was chosen for the vertical *y*-axis, to line-up compounds belonging to phenyl-specific linkages. As illustrated in Fig. 3, each data point in this 2D mass defect matrix diagram represented a specific component in the mass spectrum of the lignin decomposition mixture, and structurally related compounds were therefore quickly identified for further interrogation. To demonstrate the benefits of this 2D strategy, Fig. 3 illustrates a small region that was further expanded from the 2D plot. In this magnified view, three series of data points are highlighted with red, green and brown colours. The measured *m*/*z* value for the first (red) data point was *m*/*z* 163.0389. This precursor ion was then further analysed by CID experiments and revealed the structure to be coumaryl acid (the CID spectrum is shown in Fig. S2, ESM). The two following, related red data points on the vertical *y*-axis indicated that up to two methoxy (OCH_3_) groups can be attached to the compound. To confirm their identities, CID analyses were performed again. Although the absolute methoxylation sites could not be determined from these MS/MS experiments, it was clear that methoxylation occurred on the phenyl core of coumaryl acid and the two compounds corresponding to the red colour points were likely to be coniferyl and sinapyl acid (Fig. S2, ESM).

Similarly, on the *x*-axis, the green and brown data points were both aligned with the coumaryl alcohol series horizontally, which indicated that they shared the same core structure. CID experiments revealed that the structures of these compounds were linked by phenyl additions to the coumaryl acid core (Fig. 3, top).

In addition, it was interesting to note that there was a distinct gap with no signals in the vertical direction of Fig. 3, below the three red points belonging to the coumaryl acid series discussed above, followed by three further data points (shown by a bracket in Fig. 3). CID analysis of these additional three compounds was inconsistent with the coumaryl series, however. This phenomenon highlighted that constitutional isomers likely existed in the aligned compound series, and the gap between the points was an indication for the occurrence of such isomers of structurally different ion species.

Kattner et al. pointed out that for *m*/*z* values greater than 600, and element restrictions to C, H, O, N, P and S and a tolerance mass window of 1 ppm, more than 15 reasonable molecular formulae can be calculated [43]. Therefore, determining the correct elemental formula for lignin components from the many possible assignments is a challenging task during data processing. After plotting the data points using the rescaled axes in the 2D matrix diagram, however, structurally related compounds are lined up and readily identifiable from the thousands of different signals. Consequently, the chosen approach improved the confidence for unambiguous peak assignments, because the elemental formulae were determined by using the homologous species as a basis with strict elemental restrictions. With increasing *m*/*z* value, the number of the possible elemental formulae increased exponentially, but the 2D approach permitted determination of theoretical *m*/*z* and homologous chemical structures from genealogical links, rather than unsystematically assigning elemental compositions in a shotgun manner, thus significantly improving trust in the peak assignments.

**Fig. 4:**
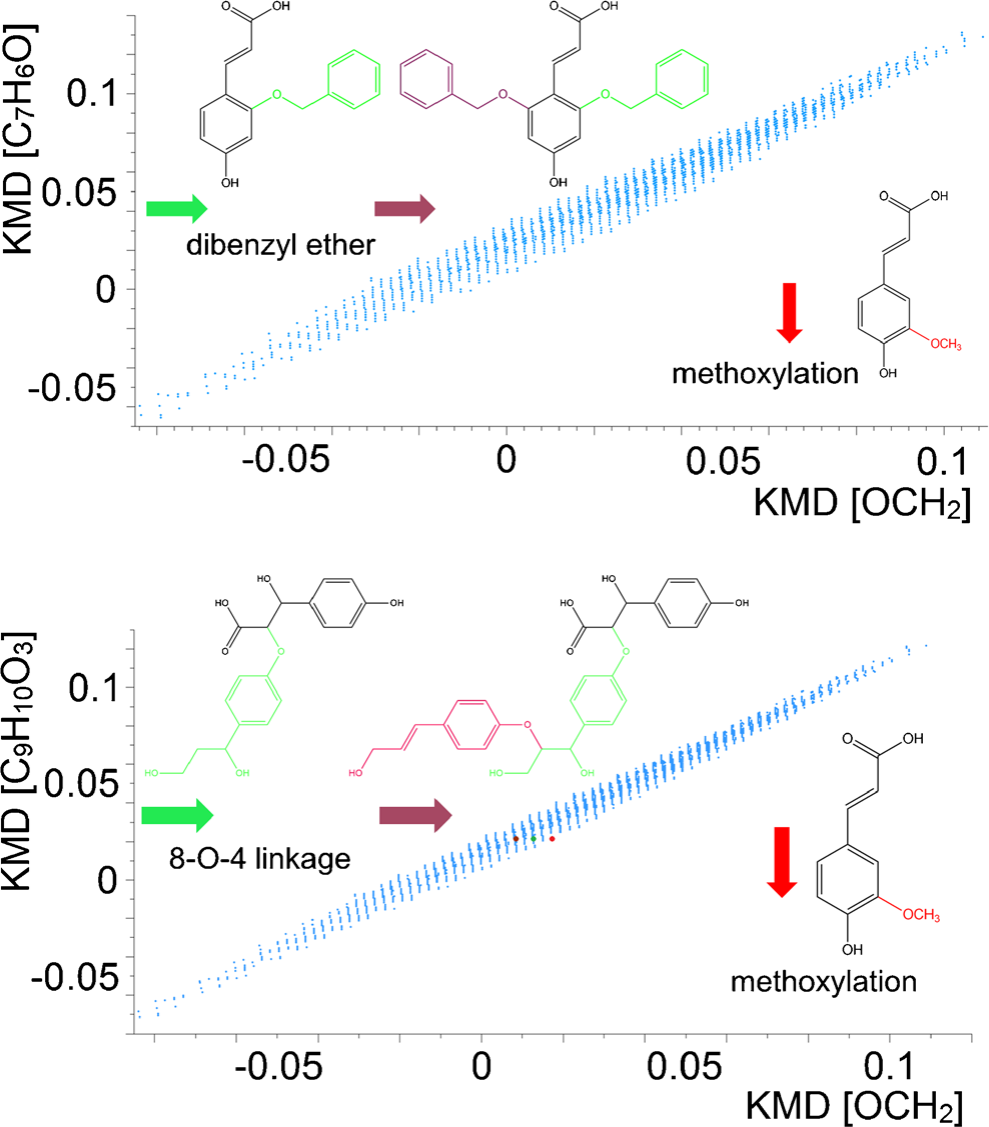
Two-dimensional mass defect matrix plot for lignin sample components. *Top*: [OCH_2_] versus [C_7_H_6_O]; *bottom*: [OCH_2_] versus [C_9_H_10_O_3_]. The *insets* show the basic structures of coumaryl acid (*black*), methoxylation (*red*), dibenzyl ether and 8-O-4 linkages (*green* and *brown*)

**Fig. 5:**
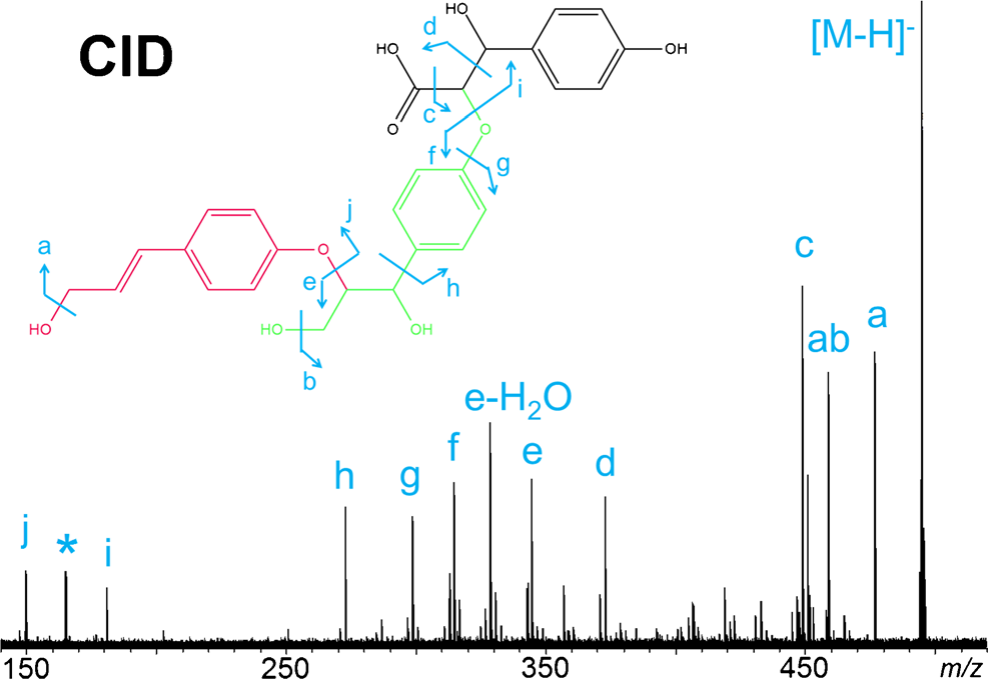
CID–MS/MS spectrum of the deprotonated [M−H]^−^ ion of C_27_H_28_O_9_, with fragments labelled in the proposed molecular structure. (Note *asterisk* labels an FTICR artefact and corresponds to the third harmonics of the precursor ion at *m*/*z* 495. The detailed peak assignment is summarised in Table S3 (ESM)

The 2D matrix plot can be further extended to other building blocks of lignin oligomers, to provide a tailored decomposition analysis of the complex mass spectra for specific connectivities. For example, the KMD bases can be modified to probe the various other lignin linkages. Typical linkages, such as dibenzyl ether and 8-O-4 linkages, were also interrogated in this study, the results of which are summarised in Fig. 4. Starting from the basic core structures in the low *m*/*z* region, the metrics not only improved the structural overview of the spectrum of lignin decomposition products but also eased the constraints for the mass accuracies needed for peak assignments in the complex mass spectra. In the mixtures, it was always difficult to pre-isolate compounds of higher molecular weights, but lower abundances for MS/MS experiments, as a result of interferences from isobaric components. The 2D matrix plots offered a complementary approach to predict the possible candidate structures. Figure 4 shows an illustrative example for the component with measured *m*/*z* at approx. 495 (red colour). The 2D matrix plot demonstrated that this data point lined up with coumaryl acid (brown data point), with two C_9_H_10_O_3_ units separating the two components. Hence, its elemental formula was determined to be C_27_H_28_O_9_. In addition, its molecular structure was also predicted according to the KMD base applied, after attaching two building blocks via the 8-O-4 linkage of coumaryl acid. Subsequent CID experiments (Fig. 5) verified that the proposed structure was indeed correct; a sole chemical structure was assigned to C_27_H_28_O_9_.

### Abundance comparisons for selected species

As shown in the previous section, the horizontal and vertical axes of the 2D matrix plots align the compounds according to the specific KMD base used for data interrogation. In addition to these parallel axes, the slopes of the slanted lines also have diagnostic potential. Reactions that involve a loss or gain of a specific elemental formula of structural-related compounds can be identified from these trend lines, as, theoretically, each reaction pathway has its own trend line with its characteristic slope and intercept.

**Fig. 6:**
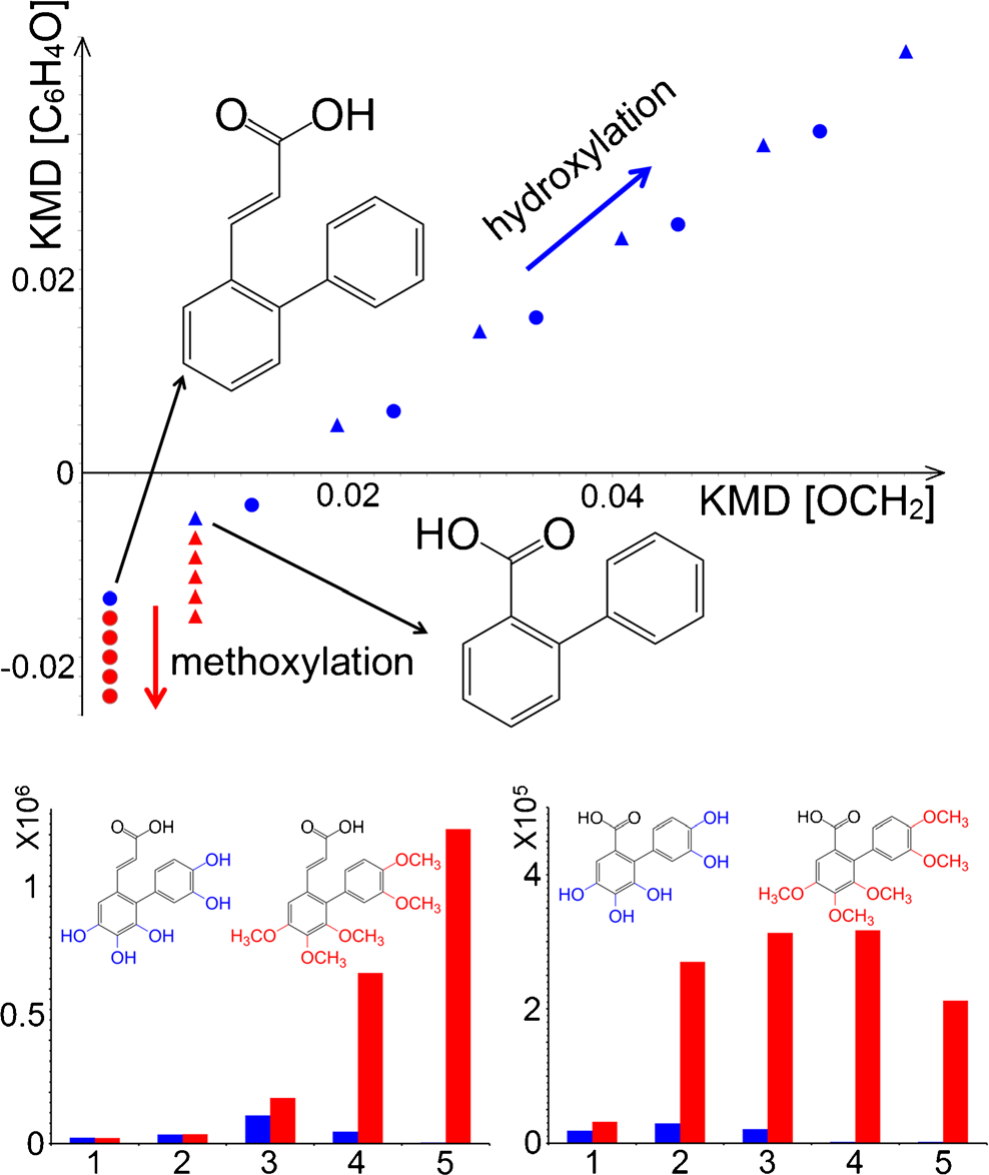
*Top* Visualisation of hydroxylation (*blue*) and methoxylation (*red*) reaction trend lines for two biphenyl compounds in the 2D matrix plot. The *circular points* represent acrylic acid-substituted biphenyls; *triangular points* correspond to biphenyl-2-carboxylic acid derivatives. *Bottom* Abundances of biphenyl compounds after hydroxylation (*blue*) or methoxylation (*red*); the *x-axis* represents the number of –OH or –OCH_3_ groups added to the compounds. Detailed compound formulae and their peak areas can be found in Table S4 (ESM)

It is apparent from Fig. 3 that numerous trend lines in the 2D matrix plot can be readily distinguished, and particular chemical decomposition reactions can be selected for further analysis. The trend lines representing the hydroxylation and methoxylation reactions for two biphenyl compounds were selected as examples here. Their relative peak abundances were compared and the results shown in Fig. 6. From the plot, it is evident that up to five functional groups were added to both compounds. In addition, methoxylation was favoured over hydroxylation for these two selected lignin decomposition products, as the methoxylation product abundances were several orders of magnitude higher than those of hydroxylation products. On the other hand, detailed comparison of these two compound series also demonstrated that the total number of products with attached acrylic acid residue in the 2-position was about fivefold higher than products with attached biphenyl-2-carboxylic groups; this was probably due to the longer side chain of acrylic acid, which provided higher spatial flexibility to the benzene ring for the additional reactions. Probing the possible decomposition pathways via trend lines is a typical analysis from the van Krevelen diagram [28]. Here, this function was greatly enhanced by plotting the data in two dimensions, by allowing the trend line approach to be applied to the 2D matrix plots of lignin products. Of course, given the complexity of the investigated samples, it is always possible that a data point within these plots consists of a mixture of constitutional isomers with different chemical structures.

While the shown strategy is not a replacement for detailed MS/MS experiments, the 2D matrix plots developed here nevertheless provide a simple filtering and data simplification strategy to reduce the complexity of the data sets, and to allow for selection of relevant precursor structures from the full-scan mass spectra for further study.

## Conclusions

High resolution mass spectrometry is widely applied in the analysis of complex samples, such as crude oil, asphaltenes, humic acids and lignin. Recent advances in HRMS have strongly increased the amount of information that can be gathered from a single mass spectrum, including detailed compositional information from spectra that contain thousands of signals. The primary full-scan mass spectrum initially only provides the elemental compositions, and great manual data processing efforts are often required to extract the required information from the data, to achieve the ultimate goal of structural identification of each of the components in the mixture. While liquid chromatography can be used to fractionate complex samples to achieve a certain degree of separation, the enormous complexity of some samples, such as those investigated here, requires further measures to aid the structural elucidation process. Because it is not feasible to conduct detailed MS/MS analyses for each of the *m*/*z* features in the sample, various post-processing methods have been developed. KMD plots are widely adopted in petroleomics studies to visually separate compounds with the same heteroatom content (e.g. O, S, N etc.) on the basis of mass defect. Lignin decomposition products, however, are composed of three elements only, viz. C, H and O. Therefore, KMD plots were modified in this study to allow classification of the different internal linkages of the lignin oligomers. The necessary rescaling of the *m*/*z* domain can be easily performed via most open source MS tools such as mMASS [44], or simply by using Excel spreadsheets. The strategy shown here quickly highlighted linkage-specific components, provided reasonable prediction of their structures and identified possible reaction pathways for further investigation. The method therefore readily facilitated peak assignment in HRMS spectra of lignin decomposition products. Importantly, the 2D matrix mapping strategy can be equally applied to other complex samples containing molecules with small numbers of heteroatoms, such as natural organic matter, asphaltenes, humic acids and naphthenic acids.

## Electronic supplementary material

Below is the link to the electronic supplementary material.

ESM 1 (PDF 717 kb)
