# Supplementary material for: Two-dimensional mass defect matrix plots for mapping genealogical links in mixtures of lignin depolymerisation products
Source: Anal Bioanal Chem. 2016 May 14;408:4835–43. doi: 10.1007/s00216-016-9598-5 (PMC4914518; doi:10.1007/s00216-016-9598-5)
Supplement: Supplementary file 1 — (PDF 717 kb) [file 216_2016_9598_MOESM1_ESM.pdf]

Analytical and Bioanalytical Chemistry

Electronic Supplementary Material

Two-dimensional mass defect matrix plots for mapping genealogical links in mixtures of lignin depolymerisation products

Yulin Qi, Rolf Hempelmann, Dietrich A. Volmer

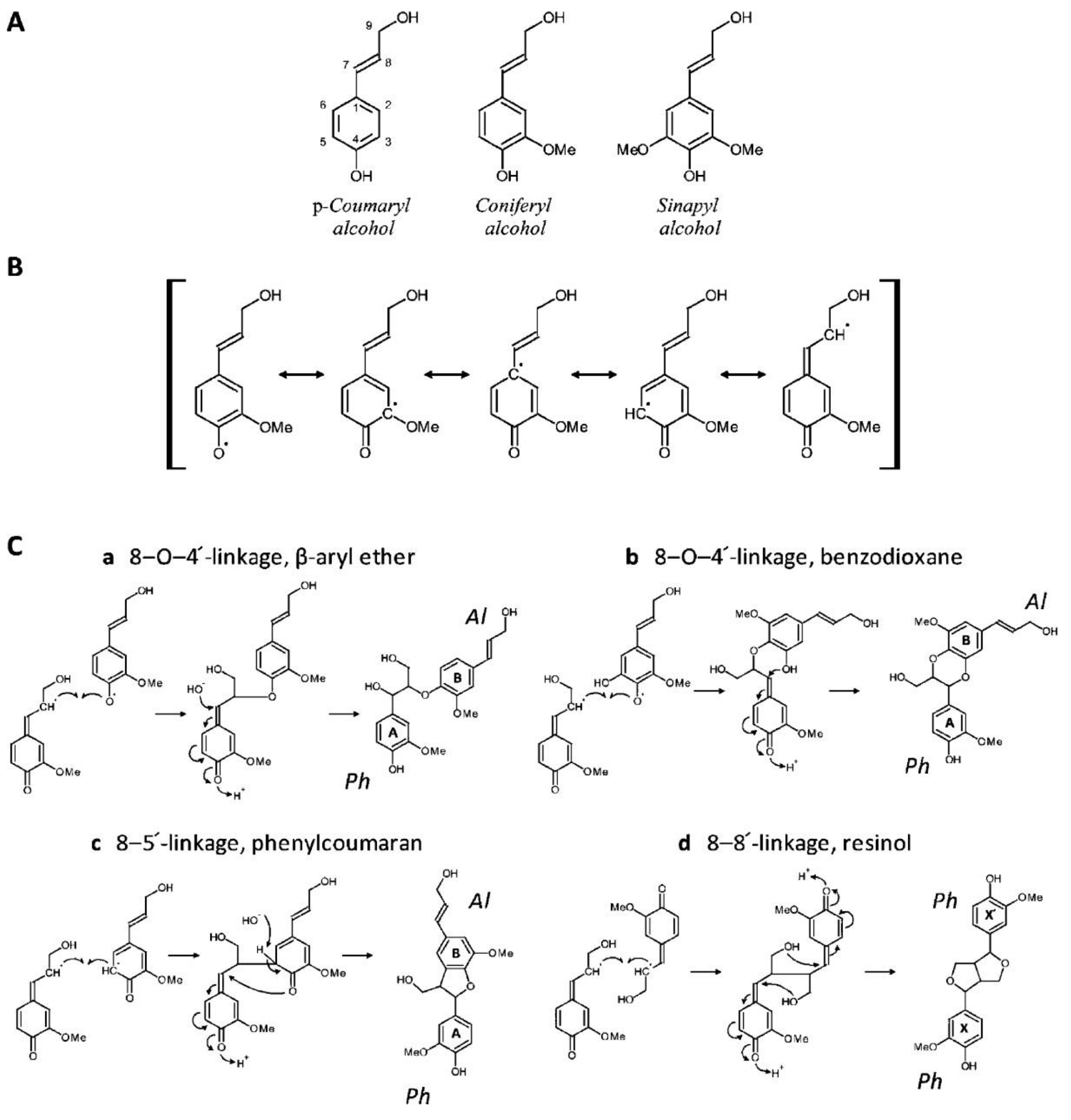

Fig. S1 Radical-radical dimerisation of monolignols. (A) Monolignols. (B) Radical delocalisation following monolignol oxidation, illustrated for the coniferyl alcohol radical. (C) Main monolignol dimerisation reactions: *Ph*, phenolic end group; *Al*, aliphatic end group. For all compounds except the resinols, the phenolic and aliphatic end groups correspond to the A and B rings, respectively. Resinols contain two phenolic end groups corresponding to the X and X’ rings. Reprinted with permission from Morreel K, Kim H, Lu F, et al (2010) Mass spectrometry-based fragmentation as an identification tool in lignomics. Anal Chem 82:8095–105

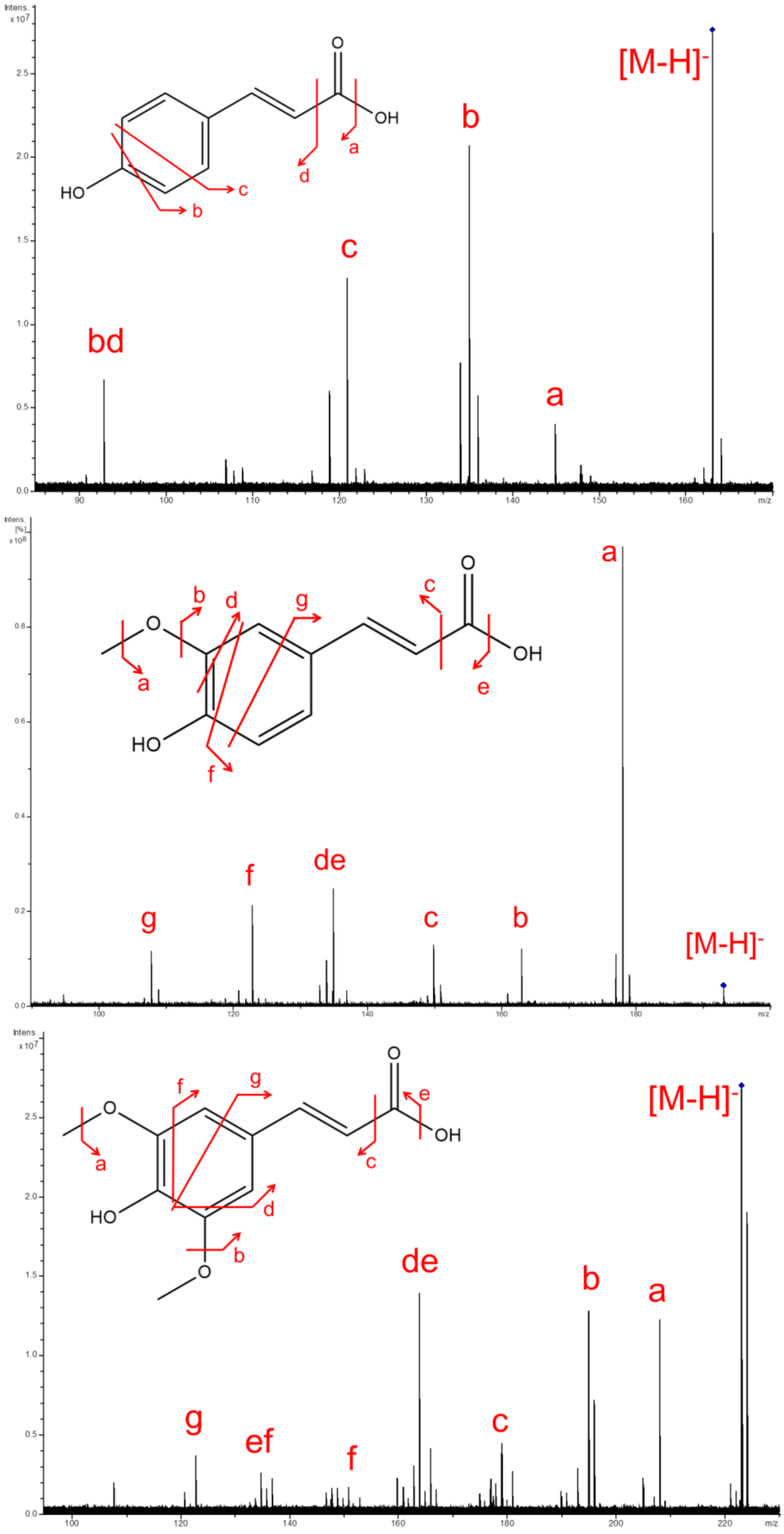

Fig. S2 CID-MS/MS spectra for the deprotonated [M-H]$^-$ ion of coumaryl (*top*), coniferyl (*middle*), and sinapyl acids (*bottom*), along with CID fragmentation schemes. The detailed peak assignments can be found in Table S2

Table S1 Peak assignments for Figure 2 (bottom)

**No.**	**Compound**	**Calc. *m/z***	**Exp. *m/z***	**mDa error**	**ppm error**
1	$C_{15}H_{16}O_{10}$	355.06707	355.066919	-0.15	-0.43
2	$C_{19}H_{16}O_7$	355.082326	355.08233	0.00	0.01
3	$C_{16}H_{20}O_7S$	355.085697	355.08572	0.02	0.06
4	$C_{16}H_{20}O_9$	355.103456	355.10336	-0.10	-0.27
5	$C_{20}H_{20}O_6$	355.118712	355.11868	-0.03	-0.09
6	$C_{23}H_{19}O_3\ ^{13}C$	355.129498	355.12973	0.23	0.65
7	$C_{17}H_{24}O_8$	355.139841	355.13984	0.00	0.00
8	$C_{21}H_{24}O_5$	355.155097	355.15505	-0.05	-0.13
9	$C_{24}H_{22}NO_2$	355.157777	355.158027	0.25	0.70
10	$C_{18}H_{28}O_7$	355.176227	355.17615	-0.08	-0.22
11	$C_{22}H_{28}O_4$	355.191483	355.19148	0.00	-0.01
12	$C_{19}H_{32}O_4S$	355.194854	355.19482	-0.03	-0.10
13	$C_{25}H_{27}O\ ^{13}C$	355.202269	355.20271	0.44	1.24
14	$C_{19}H_{32}O_6$	355.212612	355.21257	-0.04	-0.12
15	$C_{22}H_{31}O_3\ ^{13}C$	355.223398	355.22371	0.31	0.88
16	$C_{23}H_{32}O_3$	355.227868	355.22788	0.01	0.03
17	$C_{20}H_{36}O_5$	355.248998	355.24902	0.02	0.06
18	$C_{23}H_{35}O_2\ ^{13}C$	355.259784	355.26019	0.41	1.14
19	$C_{21}H_{40}O_4$	355.285383	355.28544	0.06	0.16

Table S2 CID fragments of coumaryl, coniferyl, and sinapyl acid

**coumaryl alcohol**				
**Fragment cleavage**	**Proposed formula**	**Calc. *m/z***	**Exp. *m/z***	**ppm error**
$[M-H]^-$	$C_9H_7O_3$	163.04007	163.04040	2.04
a	$C_9H_5O_2$	145.02841	145.02841	0.00
b	$C_8H_7O_2$	135.04406	135.04400	-0.41
c	$C_7H_5O_2$	121.02841	121.02830	-0.88
bd	C_6H_5O	93.03349	93.03360	1.17
**coniferyl alcohol**				
**Fragment cleavage**	**Proposed formula**	**Calc. *m/z***	**Exp. *m/z***	**ppm error**
$[M-H]^-$	$C_{10}H_9O_4$	193.05063	193.05050	-0.68
a	$C_9H_6O_4$	178.02606	178.02600	-0.34
b	$C_9H_7O_3$	163.03897	163.03890	-0.44
c	$C_8H_6O_3$	150.03115	150.03100	-0.97
de	$C_8H_7O_2$	135.04406	135.04400	-0.41
f	$C_7H_7O_2$	123.04406	123.04400	-0.46
g	$C_6H_4O_2$	108.02058	108.02050	-0.75
**sinapyl alcohol**				
**Fragment cleavage**	**Proposed formula**	**Calc. *m/z***	**Exp. *m/z***	**ppm error**
$[M-H]^-$	$C_{11}H_{11}O_5$	223.06120	223.06110	-0.43
a	$C_{10}H_8O_5$	208.03663	208.03670	0.36
b	$C_{10}H_{11}O_4$	195.06519	195.06510	-0.44
c	$C_{10}H_7O_3$	175.03897	175.03890	-0.41
de	$C_9H_8O_3$	164.04680	164.04670	-0.59
f	$C_8H_7O_3$	151.03897	151.03890	-0.47
ef	$C_8H_7O_2$	135.04406	135.04400	-0.41
g	$C_7H_7O_2$	123.04406	123.04400	-0.46

Table S3 CID fragments of compound $C_{27}H_{28}O_9$ (Figure 5)

**Fragment cleavage**	**Proposed formula**	**Calc. *m/z***	**Exp. *m/z***	**ppm error**
**[M-H]-**	$C_{27}H_{27}O_9$	495.16606	495.16600	-0.11
**a**	$C_{27}H_{25}O_8$	477.15439	477.15460	0.43
**ab**	$C_{27}H_{23}O_7$	459.14383	459.14370	-0.28
**c**	$C_{26}H_{25}O_7$	449.15948	449.15960	0.27
**d**	$C_{20}H_{21}O_7$	373.12818	373.12810	-0.21
**e**	$C_{18}H_{17}O_7$	345.09688	345.09680	-0.23
**e-H_2O**	$C_{18}H_{17}O_6$	329.10197	329.10190	-0.20
**f**	$C_{18}H_{19}O_5$	315.12270	315.12280	0.32
**g**	$C_{18}H_{19}O_4$	299.12779	299.12780	0.05
**h**	$C_{15}H_{13}O_5$	273.07575	273.07570	-0.18
**i**	$C_9H_9O_4$	181.04954	181.04960	0.36
**j**	$C_9H_9O_2$	149.05971	149.05980	0.63

Table S4 Chemical formulae and peak areas for the compounds plotted in Figure 6

blue dots ●

Compound formula	Peak area	Proposed structures
$C_{15}H_{12}O_2$	3422	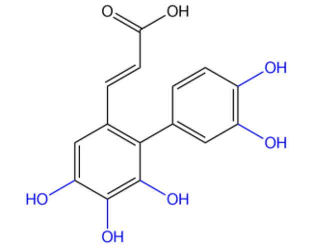
$C_{15}H_{12}O_3$	23039	
$C_{15}H_{12}O_4$	35326	
$C_{15}H_{12}O_5$	110062	
$C_{15}H_{12}O_6$	47377	
$C_{15}H_{12}O_7$	5019	

red dots ●

Compound formula	Peak area	Proposed structures
$C_{15}H_{12}O_2$	3422	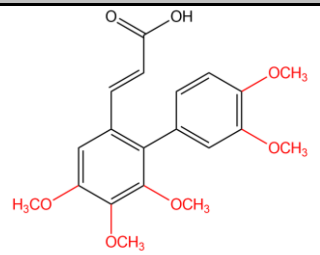
$C_{16}H_{14}O_3$	21978	
$C_{17}H_{16}O_4$	37415	
$C_{18}H_{18}O_5$	177968	
$C_{19}H_{20}O_6$	662431	
$C_{20}H_{22}O_7$	1222609	

blue triangles ▲

Compound formula	Peak area	Proposed structures
$C_{13}H_{10}O_2$	3010	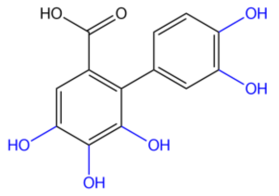
$C_{13}H_{10}O_3$	18431	
$C_{13}H_{10}O_4$	29498	
$C_{13}H_{10}O_5$	20760	
$C_{13}H_{10}O_6$	1777	
$C_{13}H_{10}O_7$	1716	

red triangles ▲

Compound formula	Peak area	Proposed structures
$C_{13}H_{10}O_2$	3010	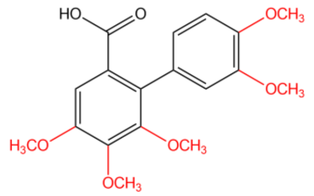
$C_{14}H_{12}O_3$	32076	
$C_{15}H_{14}O_4$	269771	
$C_{16}H_{16}O_5$	313164	
$C_{17}H_{18}O_6$	317250	
$C_{18}H_{20}O_7$	212027	
